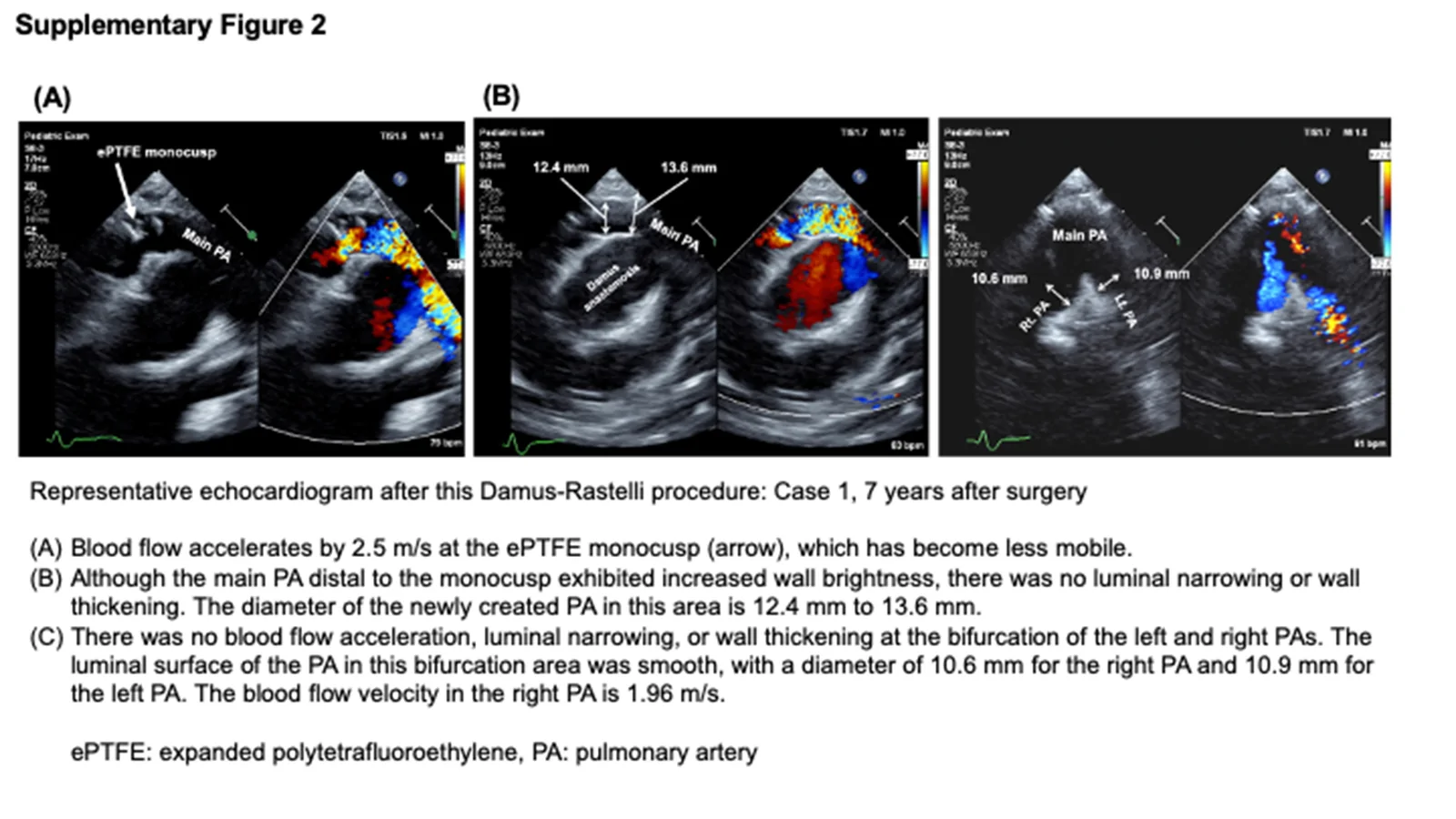# Damus-Ratelli Procedure for Double Outlet Right Ventricle With Doubly-Committed Subarterial Ventricular Septal Defect

**DOI:** 10.1016/j.atssr.2026.01.003

**Published:** 2026-01-20

**Authors:** Hayato Konishi, Akiyo Suzuki, Kanta Kishi, Takamichi Uchiyama, Takahiro Katsumata, Shintaro Nemoto

**Affiliations:** 1 Department of Thoracic and Cardiovascular Surgery, Osaka Medical and Pharmaceutical University, Takatsuki, Osaka, Japan; 2 Department of Pediatrics, Osaka Medical and Pharmaceutical University, Takatsuki, Osaka, Japan; 3 Uchiyama Kids Clinic, Takatsuki, Osaka, Japan

## Abstract

To prevent interventricular rerouting tunnel obstruction after biventricular repair for double outlet right ventricle, it is essential to create a sufficiently straight and wide interventricular rerouting. In cases of the ventricular septal defect extending under both semilunar valves arranged side by side and perpendicular to the outlet septum, conventional rerouting to one semilunar valve may obstruct the other. Herein, we describe our technique for creating an interventricular rerouting tunnel in this rare anatomical configuration by combining rerouting from ventricular septal defect to both semilunar valves and Damus anastomosis. Upon completion, the Rastelli procedure without a conduit was performed.

For successful biventricular repair of double outlet right ventricle (DORV), the creation of a sufficiently wide and straight intraventricular rerouting (IVR) tunnel from the ventricular septal defect (VSD) to either semilunar valve is essential to prevent subaortic obstruction, which is a serious complication with a high recurrence rate affecting not only cardiac function but also mortality.^1^^,^^2^ This is because discrete or diffuse pseudointimal proliferation due to turbulent flow within the insufficient IVR tunnel might cause the obstruction.^1^^,^^3^ However, there are some anatomical configurations, such as in DORV with remote noncommitted VSD or a rare form of DORV with the doubly committed subarterial VSD extending below both semilunar valves and the muscular or fibrous outlet septum (conus) locating perpendicular to the middle of the VSD crest, where it is difficult to create a sufficient IVR.^4^^,^^5^ In this report, we evaluated the midterm results of our modification, particularly a combination of IVR from the VSD to both semilunar valves and Damus anastomosis, as an alternative to the IVR reported in the latter rare condition.^6^

## Technique

The institutional review board of Osaka Medical and Pharmaceutical University approved this study (ID#2021-183, extended to April 30, 2025). Written informed consent was obtained from the patients’ parents.

**Figure 1:**
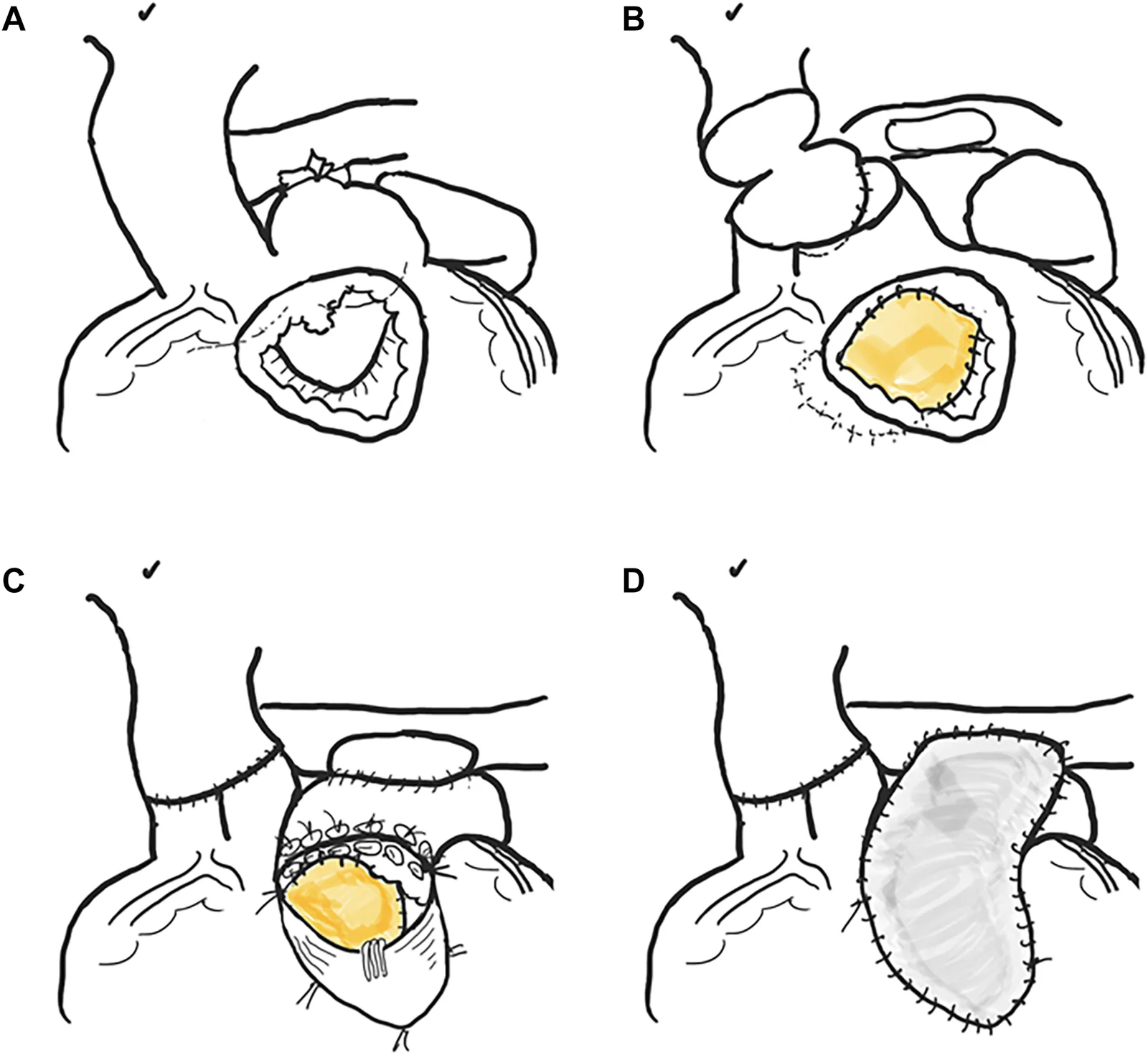
Surgical method. (A) A transverse right ventriculotomy is performed in the outlet free wall, and, if present, the distal conus septum is resected. (B) A wide patch is sutured to create a straight and wide interventricular rerouting tunnel from the ventricular septal defect to both semilunar valves. The 2 great arteries are divided at the sinotubular junction, and a double-barreled Damus anastomosis is made to drain blood flow from the 2 semilunar valves into the ascending aorta. (C) The distal stump of the main pulmonary artery is attached to the upper surface of the left appendage, and the inferior border is approximated to the upper rim of the right ventriculotomy in a normal relationship with the ascending aorta. A bullet-shaped monocusp is placed at the lower rim of the right ventriculotomy. (D) Establishment of a new Rastelli route with a transannular patch using glutaraldehyde-treated autologous pericardium.

**Figure 2:**
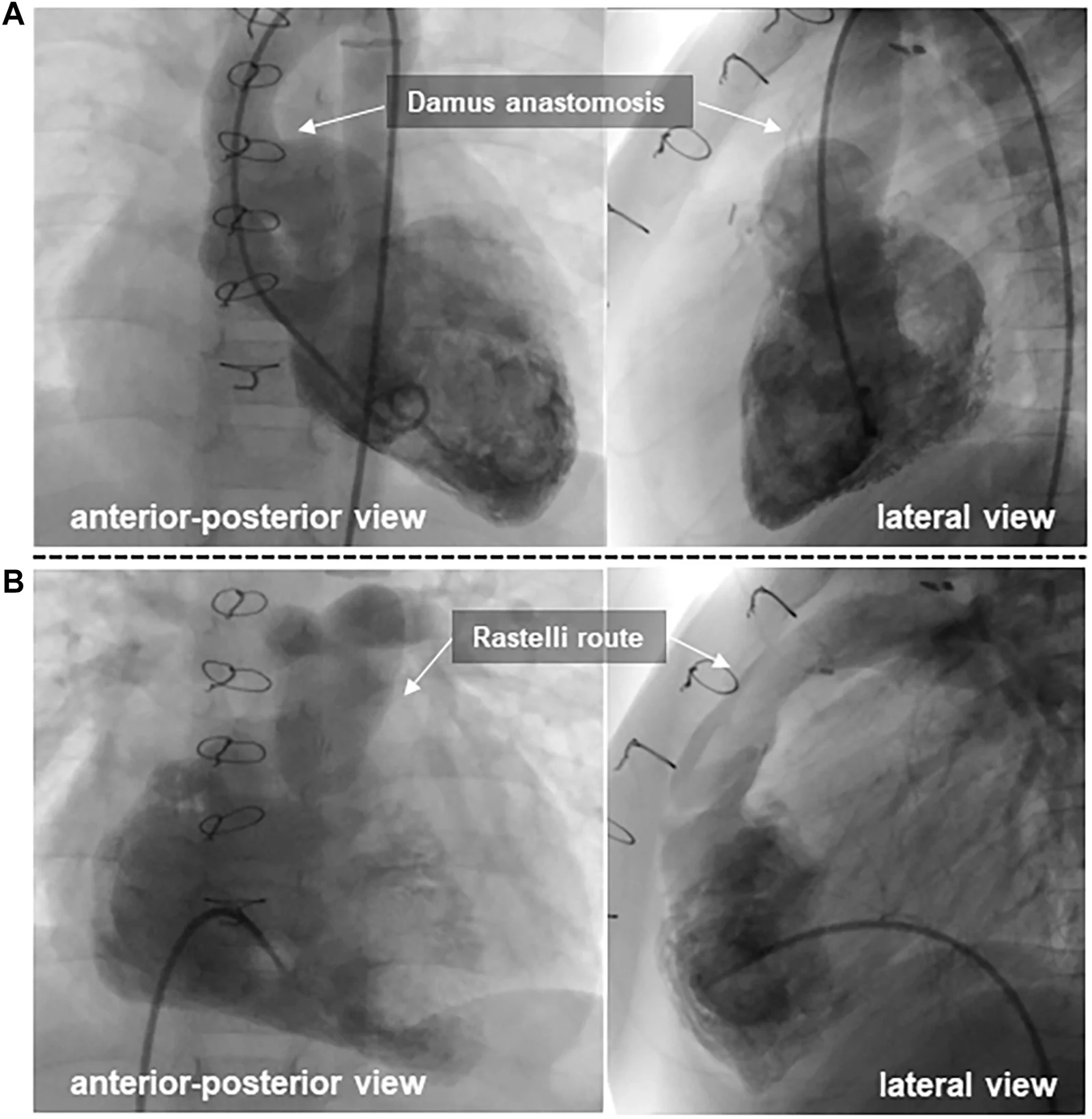
Representative (A) left and (B) right ventriculography at 5 years after the combination procedure.

**Supplementary Figure 1:**
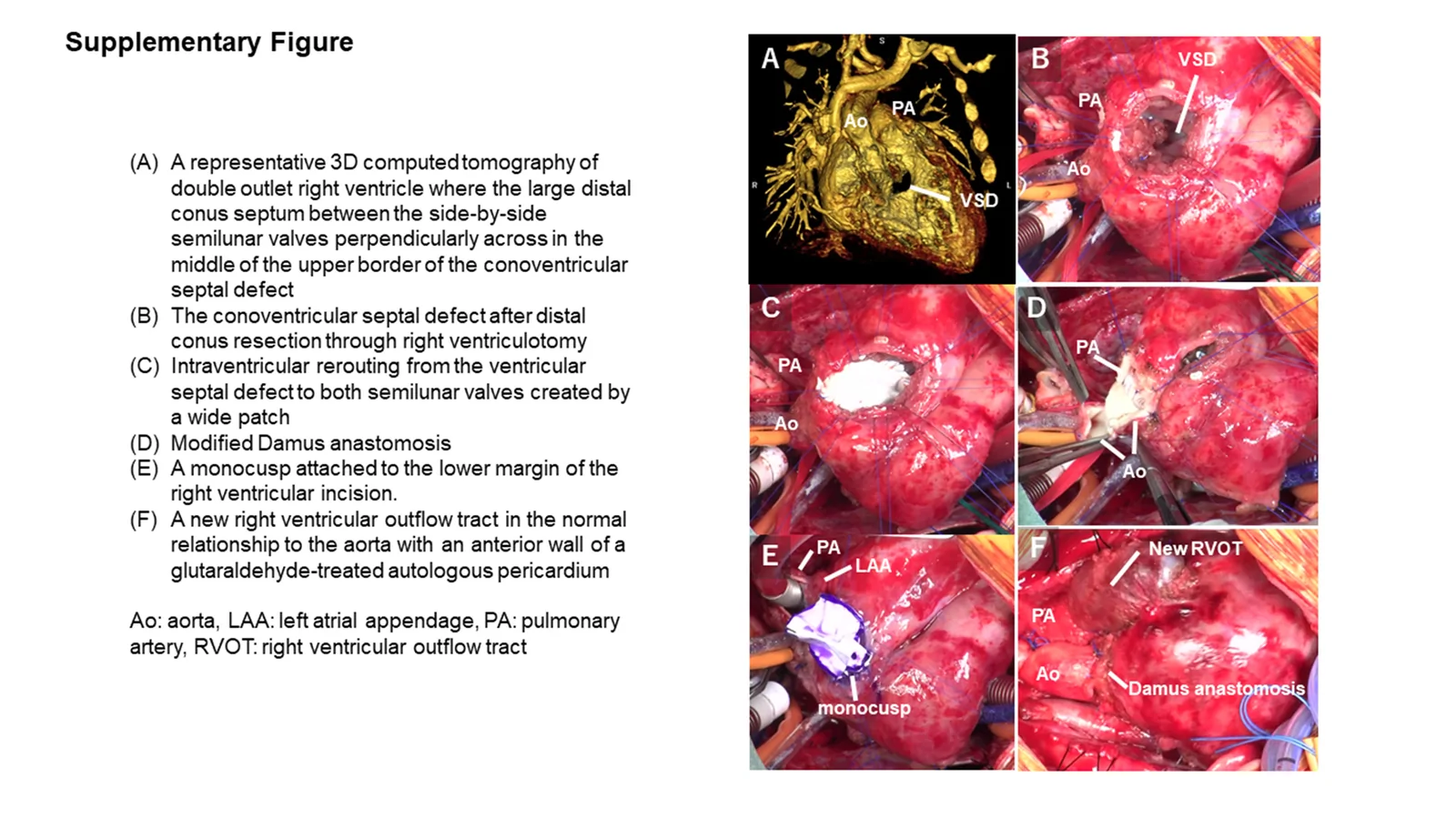


Under cardiopulmonary bypass and cardioplegic arrest, the free wall of the right ventricle (RV) outlet is incised transversely, and the muscular outlet septum is resected if present (Figure 1A). A wide expanded-polytetrafluoroethylene patch (0.4 mm, Gore & Assoc) was sutured to create a straight and wide IVR tunnel from the VSD to both semilunar valves (Figure 1B). The two great arteries are divided at the sinotubular junction, and the double-barrel type Damus anastomosis is made to drain blood flow from both semilunar valves into the ascending aorta (Figure 1B). The distal stump of the main pulmonary artery (PA) is attached to the upper surface of the left appendage, of which the inferior border is approximated to the upper rim of the RV incision to reconstruct the posterior wall of a new RV-PA route in a normal orthotopic relationship with the ascending aorta (Figure 1C). A bullet-shaped monocusp created using an expanded-polytetrafluoroethylene pericardial sheet (0.1 mm, as above) was placed at the lower rim of the RV incision (Figure 1C). A new RV-PA route was established with an anterior roof using glutaraldehyde-treated autologous pericardium (Figure 1D). Representative intraoperative images of this procedure are shown in Supplemental Figure 1. Representative postoperative angiography of both ventricles demonstrates a wide left ventricular outflow tract (Figure 2A) and a naturally curved RV-PA route (Figure 2B).

To date, this procedure has been performed in 3 consecutive patients after palliative pulmonary artery banding. The age and body weight of each patient at this repair were 10, 8, and 8 months and 5.5, 6.5, and 7.4 kg, respectively. The diameter of the new RV-PA route was 12 mm in all cases. All patients had an uneventful postoperative course with a good functional status. Echocardiography showed no flow acceleration in all IVR tunnels (1.8, 1.0, and 1.5 m/s) at 8, 6, and 5 years postoperatively in case 1, 2, and 3, respectively. Flow acceleration across the monocusp with gradually reduced mobility was 2.5, 2.4, and 1.8 m/s, respectively, with mild regurgitation. There was no PA deformity or stenosis from the anastomosed sinuses of the Damus (Figure 2, Supplemental Figure 2).

## Comment

The incidence of concordant atrioventricular connection and side-by-side great arteries with doubly committed subarterial VSD, which is the focus of this brief communication, is only 3% of DORV cases.^4^ In this anatomical configuration, the fibrous or muscular outlet septum crosses perpendicular to the center of the VSD crest, and both semilunar valves override the crest, resulting in a double ventricular hemodynamic outlet.^5^^,^^6^ Recently, a short-term case series describing a new promising IVR procedure from VSD to the aortic valve for this rare DORV has been reported.^6^ On the other hand, there might still exist a possible risk of IVR tunnel obstruction developed in the long-term due to the long and angled IVR tunnel created by the two combined patches. Therefore, our modification would be more advantageous in terms of a wide, straight, and short IVR tunnel to avoid turbulent flow. A method similar to ours has been reported for biventricular repair of Taussig-Bing anomaly where arterial switch or Kawashima-type IVR were not applicable because of coronary artery anomaly or obstructive subaortic conus, respectively.^3^^,^^7^ Therefore, this report might have shown an extended application of this method to this rare type of DORV.

In small-diameter RV-PA reconstruction, although the Rastelli procedure using a conventional small-diameter conduit frequently requires early reoperation, our reconstruction method with maximum use of autologous tissue showed good log-term durability with an estimated 5-year free reoperation rate of 92.9%.^8^

In conclusion, a combination of Damus-Rastelli procedure could be a simple but effective alternative for biventricular repair of DORV with the doubly committed subarterial VSD extending under both semilunar valves arranged side by side and perpendicular relationship to the outlet septum. Long-term follow-up and assessment are mandatory to warrant the efficacy and safety of this combination.

## Figures and Tables

**Supplementary Figure 2:**